# Patient Perspectives With Telehealth Visits in Cardiology During COVID-19: Online Patient Survey Study

**DOI:** 10.2196/25074

**Published:** 2021-01-22

**Authors:** Aniruddha Singh, Natalie Mountjoy, Doug McElroy, Shilpi Mittal, Bashar Al Hemyari, Nicholas Coffey, Kristen Miller, Kenneth Gaines

**Affiliations:** 1 Western Kentucky Heart and Lung Research Foundation Bowling Green, KY United States; 2 Western Kentucky University Bowling Green, KY United States; 3 Vanderbilt University Medical Center Nashville, TN United States; 4 University of Kentucky College of Medicine Bowling Green, KY United States

**Keywords:** COVID-19, telehealth, cardiology, internet, broadband, patient satisfaction, restriction, survey

## Abstract

**Background:**

The rise of COVID-19 and the issue of a mandatory stay-at-home order in March 2020 led to the use of a direct-to-consumer model for cardiology telehealth in Kentucky. Kentucky has poor health outcomes and limited broadband connectivity. Given these and other practice-specific constraints, the region serves as a unique context to explore the efficacy of telehealth in cardiology.

**Objective:**

This study aims to determine the limitations of telehealth accessibility, patient satisfaction with telehealth relative to in-person visits, and the perceived advantages and disadvantages to telehealth. Our intent was two-fold. First, we wanted to conduct a rapid postassessment of the mandated overhaul of the health care delivery system, focusing on a representative specialty field, and how it was affecting patients. Second, we intend to use our findings to make suggestions about the future application of a telehealth model in specialty fields such as cardiology.

**Methods:**

We constructed an online survey in Qualtrics following the Patient Assessment of Communication During Telemedicine, a patient self-report questionnaire that has been previously developed and validated. We invited all patients who had a visit scheduled during the COVID-19 telehealth-only time frame to participate. Questions included factors for declining telehealth, patient satisfaction ratings of telehealth and in-person visits, and perceived advantages and disadvantages associated with telehealth. We also used electronic medical records to collect no-show data for in-person versus telehealth visits to check for nonresponse bias.

**Results:**

A total of 224 respondents began our survey (11% of our sample of 2019 patients). Our recruitment rate was 86% (n=193) and our completion rate was 62% (n=120). The no-show rate for telehealth visits (345/2019, 17%) was nearly identical to the typical no-show rate for in-person appointments. Among the 32 respondents who declined a telehealth visit, 20 (63%) cited not being aware of their appointment as a primary factor, and 15 (47%) respondents cited their opinion that a telehealth appointment was not medically necessary as at least somewhat of a factor in their decision. Both in-person and telehealth were viewed favorably, but in-person was rated higher across all domains of patient satisfaction. The only significantly lower mean score for telehealth (3.7 vs 4.2, *P*=.007) was in the clinical competence domain. Reduced travel time, lower visit wait time, and cost savings were seen as big advantages. Poor internet connectivity was rated as at least somewhat of a factor by 33.0% (35/106) of respondents.

**Conclusions:**

This study takes advantage of the natural experiment provided by the COVID-19 pandemic to assess the efficacy of telehealth in cardiology. Patterns of satisfaction are consistent across modalities and show that telehealth appears to be a viable alternative to in-person appointments. However, we found evidence that scheduling of telehealth visits may be problematic and needs additional attention. Additionally, we include a note of caution that patient satisfaction with telehealth may be artificially inflated during COVID-19 due to external health concerns connected with in-person visits.

## Introduction

In its most simplistic form, telehealth or telemedicine refers to the mixture of art and science to maintain health and prevent disease from a distance [1]. The definition of telehealth has evolved along with technological advances. Medicaid currently defines it as “two-way, real time interactive communication between the patient, and the physician or practitioner at the distant site. This electronic communication means the use of interactive telecommunications equipment that includes, at a minimum, audio and video equipment” [2,3]. The use of telehealth has been increasing, as demonstrated by the rise of telehealth visits among the commercially insured from 206 (0.02 per 1000) in 2005 to 202,374 (6.57 per 1000) in 2017. This annual growth rate of 52%, and the 261% increase between 2015-2017 alone, is likely associated with the rise of parity laws mandating coverage for such visits. The main contributors to this rise have been in primary care telehealth and tele–mental health visits [4]. The medium has been adopted by disciplines that require minimum physical exam findings, such as radiology and dermatology, while other, more heavily exam-dependent, specialties such as cardiology have been more resistant [5].

Perceived barriers from the physician-side include the lack of a comprehensive physical examination, technically challenged staff and patients, public resistance to telehealth, cost, reimbursement issues, and lower standards of care concerns [5,6]. Naser et al [7] conducted a literature review to present perspectives of telemedicine in cardiology in Bosnia and Herzegovina. This study provided an interesting take on how telemedicine is advancing in transitional countries and focused mainly on the different types of technology needed for a patient encounter. The key issue seemed to be development of software that would provide authentic data and be available for patients’ use. The authors suggested a primary limitation on the use of telemedicine, or information technology itself in medicine, was poor quality of software solutions and poor connectivity, with inadequate software maintenance. Although these and other technological factors can limit its use in rural areas, Naser et al [7] concluded that interactive video consultations provided better access to heart specialists and subspecialists than other means, accurate diagnosis, better treatment, reduction of mortality, and a significant reduction in costs.

Additionally, Di Lenarda et al [8] examined the strategic importance of innovative models of care for nonhospitalized patients with heart failure, along with the challenges and opportunities for its widespread clinical implementation. Their research revealed that technology development is mostly market driven, leading to an excess of data, unverifiable quality, and scarce utility. They recommended a multidisciplinary and multi-professional “Chronic Care Model” of integration between hospital and territory, and suggested that Italy’s active role in integrating telemedicine is helping to avoid heart failure hospitalizations.

Despite the continuing dialectic around the efficacy of telehealth in cardiology, the onset of the global COVID-19 pandemic necessitated a more or less immediate shift toward remote modalities to ensure continuation of care for cardiology patients, without increasing health risks. The transition has generated many important research questions about not only quality care but also patient use and perceptions of the novel modality. Will patients be able to access this care? Will they be satisfied with the experience? What are their perceived advantages and disadvantages to this new approach? Few studies have evaluated satisfaction with telemedicine in a broad range of cardiology patients, but what is available comes mostly from heart failure studies. Kraii et al [9] evaluated 14 publications from multiple databases. They found patients were satisfied with telemedicine but that the measurement of patient-reported outcomes, such as patient satisfaction with noninvasive telemedicine in patients with heart failure, is underexposed. None of the studies examined provided a clear definition or concept of patient satisfaction with telemedicine, and all studies evaluated patient satisfaction using different scales or questionnaires. The authors recommended that patient satisfaction become a more prominent theme in telemedicine research and that well-designed, validated, and standardized instruments with theoretic foundations were needed to measure patient satisfaction with telemedicine.

One such instrument, developed and validated by Agha et al [10], is a self-report questionnaire called the Patient Assessment of Communication During Telemedicine (PACT). The PACT is built on the four key domains of the physician-patient experience: patient-centered communication, clinical competence, interpersonal skills, and supportive environment [11-13]. The domain of “patient-centered communication” assesses the perception of the physicians’ active involvement with patients. Items regarding the “perceived clinical competence” of the physician focus on the patient’s experience with the clinical examination and their confidence in the physician’s clinical abilities. Patient perception of “interpersonal skills” includes patient’s emotional needs and comfort in discussing medical concerns with their providers. The “supportive environment” domain measures patients’ perception of professionalism with their cardiologist and other in-office personnel. The theoretical foundations of instruments like the PACT allow for a comparison between patients’ perceptions of telehealth visits and standard in-person visits; the four domains are transferable to both modalities.

Kentucky presently serves as an ideal study location in the United States for examining the efficacy of and patient satisfaction with telehealth in cardiology. In recent years, Kentucky has ranked in the top 10 states for prevalence of obesity (2018) and among the top five states for prevalence of diabetes (2016) [14,15]. These factors contributed to the state’s top 10 ranking in age-adjusted total cardiovascular deaths per 100,000 persons (from 2016 to 2018) [16]. This poor chronic health standing is compounded by the fact that Kentucky ranks in the bottom 10 states for household income as of 2018 [17]. Economic constraints combined with rural geography contribute to a lack of internet availability; one of every four households in Kentucky lacks a broadband internet connection [18]. However, the rise of COVID-19 and issuance of a mandatory stay-at-home order for all nonessential employees by the Kentucky State government on March 16, 2020, necessitated use of a direct-to-consumer model for cardiology telehealth for adult patients.

The cardiovascular needs of Kentuckians, coupled with the limitations described, provides the context for a timely natural experiment. Here, we use a survey of cardiology patients to investigate the utility of telehealth from their perspective. Our primary objectives were to determine the existing limitations of telehealth accessibility, patient satisfaction with telehealth relative to traditional in-person visits in a situation where the mandatory shift to telehealth minimized self-selection bias, and the resulting perceived advantages and disadvantages to telehealth. Our intent was two-fold. First, we wanted to conduct a rapid postassessment of the mandated overhaul of the health care delivery system, focusing on a representative specialty field, and how it was affecting patients. We needed to know what was working and what was not so as to inform adaptive management in the near term. Second, we intended to use our findings to make suggestions about the future application of a telehealth model in specialty fields such as cardiology.

## Methods

We employed a web-based survey and used existing electronic medical record (EMR) data to answer these research questions. Although an online survey may seem like an odd choice (the same barriers that may keep patients from using telehealth could also keep them from answering an online survey on a PC or other device, such as lack of broadband internet access or lack of computer skills), it afforded the rapid analyses required to answer these questions in real time.

### Survey Sample

We intended to survey individuals who had appointments scheduled with their cardiologist at Western Kentucky Heart and Lung (WKHL) during the COVID-19 pandemic. WKHL is the primary cardiology and pulmonary and critical care training site for the University of Kentucky cardiovascular fellowship programs in Bowling Green, Kentucky and is associated with The Medical Center as its main hospital. WKHL office staff consolidated the contact information for all patients scheduled between March 15, 2020 (the start of telehealth-only appointments due to COVID-19), and the survey implementation date on June 7, 2020. The resulting pool consisted of 2019 patients across 7 cardiologists. Our research protocol and questionnaire were approved by the Institutional Review Board of the Medical Center (IRB #20-6-05-SinA-TeleCOVID). All respondents provided an informed consent and data were kept on a secure device.

We constructed the questionnaire using Qualtrics (Qualtrics International Inc) and sent a bulk invitation email with a direct link to the online questionnaire to all 2019 patients. We optimized the survey for mobile browsers and sent two reminders, both as text messages and emails, with a direct link to the questionnaire [19]. These reminders were sent after the first week and the day the survey closed. To increase participation, we informed invitees that we would donate to COVID-19 relief efforts at The Medical Center for each completed survey [20,21]. We also provided an assurance of confidentially and included a statement of thanks to others that had responded in the reminder messages [22].

### Survey Instrument

Data were collected via an anonymous online survey following Dillman et al’s [20] Tailored Design Method for internet surveys and included expert review by cardiologists using telehealth and was pilot-testing among 25 WKHL office staff and medical interns for validity. Our questionnaire closely followed the PACT, the patient self-report questionnaire developed and validated by Agha et al [10]. As with the PACT and other studies our questionnaire assessed perspectives across the four domains of patient satisfaction: patient-centered communication, perceived clinical competence, interpersonal skills, and a supportive environment [23,24]. Aside from a few additions to address the current context of COVID-19, the accessibility of specific telehealth modalities offered, and perceived advantages and disadvantages of telehealth, all questions and items were designed based on the PACT and other validated patient surveys regarding telehealth [25]. All questions, aside from the open response, race and ethnicity, and gender, required a response for the participant to continue. Respondents were not allowed to “go back” or review their answer choices at the end of the questionnaire. Excluding consent, the questionnaire was three pages long for patients who did not have a telehealth visit and four pages long for those who did. Each page had from 7 to 24 question items (in three blocks).

Following consent, the survey began with demographic questions to ensure we could measure representation in our sample, especially because economic and health disparities may be related to demography as well as access to telehealth. Respondents were also asked if they had sought medical care during the pandemic, about their travel time to their cardiologist, and if they participated in telehealth through their cardiologist during the pandemic. The answer to this last question bifurcated respondents onto two different survey paths.

If a respondent answered “no” regarding their participation in telehealth, they were directed to a “No Tele” set of questions regarding potential barriers to their access of telehealth. They were asked what factors may have influenced their decision not to participate in a telehealth visit, which included not medically necessary, no access to a smartphone or other device, privacy concerns, preference for in-person visits, and an open response option to include other influential factors. Respondents were asked to rank each option on a 3-point Likert-type scale as not a factor, somewhat of a factor, or the primary factor.

If a respondent answered “yes” regarding their participation in telehealth, they were directed to a “Had Telehealth” set of questions. They were asked about the modality of their telehealth visit (eg, phone call or face-to-face with a smartphone, computer, or tablet) and which platform was used (eg, Zoom [Zoom Video Communications] or Doxy.me). Respondents were then asked to rank potential disadvantages (eg, technology issues due to internet connectivity, technology issues related to a device, understanding of device use, comfort communicating via camera and microphone, and privacy concerns) and potential advantages (eg, reduced travel time, reduced visit wait time, and reduced travel costs) associated with telehealth on a 3-point Likert-type scale. They were also provided an open response option to include and rank additional disadvantages and advantages. Respondents were next asked to rank their level of agreement, on a 5-point Likert scale, with 11 positive statements regarding the four domains of patient satisfaction. Lastly, respondents were asked to rank their overall experience on a 5-point Likert-type smile scale [26].

Following these two separate paths, all respondents concluded the survey with a section regarding perceptions of their standard in-person visits with their cardiologists. The first section asked respondents to rank their level of agreement, on a 5-point Likert scale, with the same 11 positive statements regarding the four domains of patient satisfaction. Similarly, they were also asked to rank their overall experience on a 5-point Likert-type smile scale [26]. Lastly, respondents were asked in an open response question if they wanted to add any other comments. They were asked to select their physician’s name from a drop-down box and were asked if they would use telehealth after social distancing measures were no longer in place.

### Electronic Medical Record Data

Aside from data collection via the survey, we also used EMR data to determine the no-show rate for telehealth appointments during our research period as well as the standard no-show rate for in-person visits during the 10 weeks prior to the state stay-at-home order. These additional data were collected to help address our questions around access to care and to ensure our sample was representative (ie, that we received enough responses from those who declined or missed their telehealth visits) and not suffering from nonresponse bias.

### Statistical Analysis

All statistical analyses were carried out using SYSTAT, version 13 (Systat Software Inc). Cronbach alpha was used to test for internal consistency and scale reliability among related questions. Paired difference in the average ratings for telehealth versus in-person appointments was tested for significance using a Wilcoxon signed rank test. Differences among cardiologists in mean ratings for telehealth versus in-person appointments were examined using Kruskal-Wallis (KW) nonparametric analysis of variance. Individual items were tested for significant differences in ratings using chi-square tests of association. Correlations among survey items were computed using Spearman rank correlations and interpreted for significance based on Bonferroni-adjusted criteria. Post hoc power analysis was used to determine the level of statistical power in our comparisons of satisfaction ratings between telehealth and in-person visits.

## Results

### Respondent Characteristics

A total of 224 unique individuals (based on Internet Protocol addresses) consented to take the survey (11% of our total sample of 2019). Of those, 86% (n=193) were recruited (ie, completed the first page and consented). Of those recruited, our completion rate was 62% (n=120), and early terminated surveys were analyzed by completed sections only. The vast majority of the 193 respondents identified as White, non-Hispanic (n=172, 89.1%); 10 (5.2%) respondents identified as African American, 2 (1.0%) as Hispanic/Latinx, 1 (0.5%) as Asian, and the remainder as unidentified; these percentages are consistent with the racial and ethnic diversity of the surrounding region [27]. The majority (n=190, 98.5%) described themselves as native English speakers. Respondents ranged from 18 to 100 years of age, with an average of 59.9 (SD 1.0) years. More than one-quarter (n=53, 27.5%) of individuals had sought medical care during the survey period; of these, slightly less than half (10.9%) did so for heart-related issues. Respondents reported a mean travel time to in-person appointments of nearly 40 minutes (mean 39.2, SE 2.5), with 9 (4.7%) indicating a 2- to 3-hour required commitment.

### Access: Reasons for Declining Telehealth

Over the course of our study period, the no-show rate for scheduled telehealth appointments at WKHL was 17% (343/2019); the no-show rate of in-person visits in the 10 weeks prior to the switch to telehealth was also between 16% and 17% (526/3172). Among our 193 respondents, 28% (n=55) did not attend their scheduled telehealth visit. However, of the 32 respondents completing the section on barriers to telehealth, 20 (62.5%) indicted they did not realize they had been scheduled for a telehealth visit during the study time frame. There were 15 (47%) respondents that cited their opinion that a telehealth appointment was not medically necessary as at least somewhat of a factor in their decision; 20 (62.5%) cited a preference for in-person appointments as at least somewhat of a factor in their declining telehealth; 7 (21.9%) cited comfort with technology as playing a role in their decision, while a small percentage identified access to technology (n=2, 6.2%) or privacy concerns (n=2, 6.2%) as factors. These data are summarized in Table 1. Additional responses collected via open response included concerns about the validity of telehealth appointments to address cardiac conditions.

**Table 1:** Distribution of responses to survey items relating to respondents’ basis for opting out of telehealth and perceived advantages/disadvantages of telehealth by those who had a telehealth appointment.

Survey items		No factor, n (%)	Somewhat, n (%)	Primary, n (%)
**Factors in declining telehealth (n=32)**				
	Not scheduled	5 (15.6)	7 (21.9)	20 (62.5)
	Not medically necessary	17 (53.1)	5 (15.6)	10 (31.3)
	Access to technology	30 (93.8)	0 (0.0)	2 (6.2)
	Comfort with technology	25 (78.1)	5 (15.6)	2 (6.3)
	Privacy concerns	30 (93.8)	1 (3.1)	1 (3.1)
	Preference for in-person	12 (37.5)	13 (40.6)	7 (21.9)
**Advantages to participating in telehealth (n=106)**				
	Reduced travel time	12 (11.3)	33 (31.1)	61 (57.5)
	Reduced visit wait time	12 (11.3)	37 (34.9)	57 (53.8)
	Travel or cost savings	19 (18.0)	44 (41.5)	43 (40.5)
**Disadvantages to telehealth (n=106)**				
	Poor internet connectivity	71 (67.0)	27 (25.5)	8 (7.5)
	Device technology issues	82 (77.4)	19 (17.9)	5 (4.7)
	Comfort with device/software	76 (71.7)	21 (19.8)	9 (8.5)
	Communication issues	73 (68.9)	26 (24.5)	7 (6.6)
	Privacy concerns	91 (85.8)	11 (10.4)	4 (3.8)

### Patient Satisfaction: Telehealth Versus In-Person Visits

Both in-person and telehealth experiences were viewed favorably, but in-person more so. The highest ratings were seen on individual items relating to the cardiologist’s perceived competence, interpersonal skills, and interest in their patient’s medical concerns; this pattern was consistent across both telehealth and in-person formats. The lowest ratings were given on items relating to the cardiologist’s support for the patient’s emotions, perceived interest in establishing a medical partnership, and thoroughness of the clinical exam. Mean scores were nearly identical among three of the four survey domains, ranging between 4.32 and 4.33 out of 5. Only the clinical competence domain generated a lower mean score (4.23), and this was driven entirely by the low rating on the item related to the thoroughness of the clinical exam; when this item was excluded, the domain mean score improved to 4.33. There was also high reliability among items within each survey domain, as Cronbach alpha values ranged from .879 to .973. These data are summarized in Table 2.

**Table 2:** Summary of responses by those who participated in telehealth, characterizing their telehealth (n=106) and in-person (n=96) experiences.

Survey domains, items, and mode			Strongly disagree, n (%)^a^	Disagree, n (%)^a^	Neither, n (%)^a^	Agree, n (%)^a^	Strongly agree, n (%)^a^	Mean (SE)^b^	*r* ^c^	*P* value^b^
**Patient-centered communication^d^**										
	**PCC^e^-1. My cardiologist seemed interested in my medical concerns.**									.74
		Tele^f^	5 (4.7)	1 (0.9)	8 (7.6)	30 (28.3)	62 (58.5)	4.35 (0.10)	0.46	
		In-P^g^	2 (2.1)	2 (2.1)	5 (5.2)	29 (30.2)	58 (60.4)	4.45 (0.09)	0.49	
	**PCC-2. My cardiologist tried to find out everything that was concerning me.**									.22
		Tele	7 (6.6)	2 (1.9)	5 (4.7)	38 (35.8)	54 (51.0)	4.23 (0.11)	0.40	
		In-P	2 (2.1)	3 (3.1)	7 (7.3)	25 (26.0)	59 (61.5)	4.42 (0.09)	0.48	
	**PCC-3. My cardiologist was interested in establishing a medical partnership.**									.16
		Tele	4 (3.8)	2 (1.9)	15 (14.1)	45 (42.5)	40 (37.7)	4.09 (0.09)	0.35	
		In-P	2 (2.1)	1 (1.0)	7 (7.3)	34 (35.4)	52 (54.2)	4.39 (0.09)	0.41	
	**PCC-4. Instructions and treatment plans were clear to me at the end of the visit.**									.54
		Tele	4 (3.8)	3 (2.8)	8 (7.6)	44 (41.5)	47 (44.3)	4.20 (0.09)	0.40	
		In-P	2 (2.1)	2 (2.1)	5 (5.2)	33 (34.3)	54 (56.3)	4.41 (0.09)	0.56	
**Clinical competence^h^**										
	**CC^i^-1. My cardiologist provided an appropriate level of medical care.**									.71
		Tele	5 (5.7)	2 (1.9)	8 (7.6)	39 (36.7)	51 (48.1)	4.20 (0.10)	0.43	
		In-P	2 (2.1)	2 (2.1)	5 (5.2)	33 (34.4)	54 (56.2)	4.41 (0.09)	0.40	
	**CC-2. My clinical exam was thorough.**									.007
		Tele	5 (4.7)	6 (5.7)	29 (27.4)	38 (35.8)	28 (26.4)	3.74 (0.10)	0.49	
		In-P	2 (2.1)	2 (2.1)	14 (14.6)	30 (31.2)	48 (50.0)	4.25 (0.10)	0.41	
	**CC-3. I had confidence in my cardiologist’s clinical competence.**									.27
		Tele	5 (4.7)	0 (0.0)	6 (5.7)	39 (36.8)	56 (52.8)	4.30 (0.09)	0.40	
		In-P	2 (2.1)	1 (1.0)	4 (4.2)	26 (27.1)	63 (65.6)	4.53 (0.08)	0.45	
**Interpersonal skills^j^**										
	**IS^k^-1. My cardiologist seemed supportive of my emotions.**									.76
		Tele	4 (3.8)	2 (1.9)	14 (13.2)	42 (39.6)	44 (41.5)	4.13 (0.10)	0.39	
		In-P	2 (2.1)	2 (2.1)	10 (10.4)	34 (35.4)	48 (50.0)	4.29 (0.09)	0.41	
	**IS-2. I was comfortable discussing my medical concerns.**									.54
		Tele	4 (3.8)	3 (2.8)	7 (6.6)	40 (37.7)	52 (49.1)	4.26 (0.10)	0.39	
		In-P	2 (2.1)	3 (3.1)	6 (6.3)	27 (28.1)	58 (60.4)	4.42 (0.09)	0.42	
	**IS-3. My cardiologist displayed appropriate interpersonal skills.**									.33
		Tele	4 (3.8)	0 (0.0)	6 (5.7)	37 (34.9)	59 (55.6)	4.38 (0.09)	0.42	
		In-P	3 (3.1)	2 (2.1)	5 (5.2)	24 (25.0)	62 (64.6)	4.46 (0.10)	0.53	
**Supportive environment**										
	**SE^l^. My interaction with other in-office personnel was professional.**									.37
		Tele	5 (4.7)	0 (0.0)	10 (9.4)	43 (40.6)	48 (45.3)	4.22 (0.09)	0.32	
		In-P	2 (2.1)	1 (1.0)	7 (7.3)	32 (33.3)	54 (56.3)	4.41 (0.09)	0.41	
**Overall**										
	**Average ratings of all items**									.001
		Tele	N/A^m^	N/A	N/A	N/A	N/A	4.19 (0.08)	N/A	
		In-P	N/A	N/A	N/A	N/A	N/A	4.40 (0.08)	N/A	
	**Overall, how did you feel about your experience?**									.22
		Tele	2 (1.9)	3 (2.8)	5 (4.7)	25 (23.6)	71 (67.0)	4.51 (0.08)	N/A	
		In-P	1 (1.0)	1 (1.0)	6 (6.4)	20 (20.8)	68 (70.8)	4.59 (0.08)	N/A	

^a^These columns show number and percentage of respondents selecting a given response.

^b^These columns summarize tests of difference in means for items between formats.

^c^This column shows the Spearman correlation between individual items and respondent’s overall rating of their experiences; all were significant at *P*<.001. Post hoc power analysis yielded levels of power >0.95 for all comparisons of individual survey items.

^d^Cronbach alpha: Tele .920 and In-*P* .973.

^e^PCC: patient-centered communication.

^f^Tele: telehealth.

^g^In-P: in-person.

^h^Cronbach alpha: Tele .879 and In-*P* .938.

^i^CC: clinical competence.

^j^Cronbach alpha: Tele .931 and In-*P* .927.

^k^IS: interpersonal skills.

^l^SE: supportive environment.

^m^N/A: not applicable.

**Figure 1:**
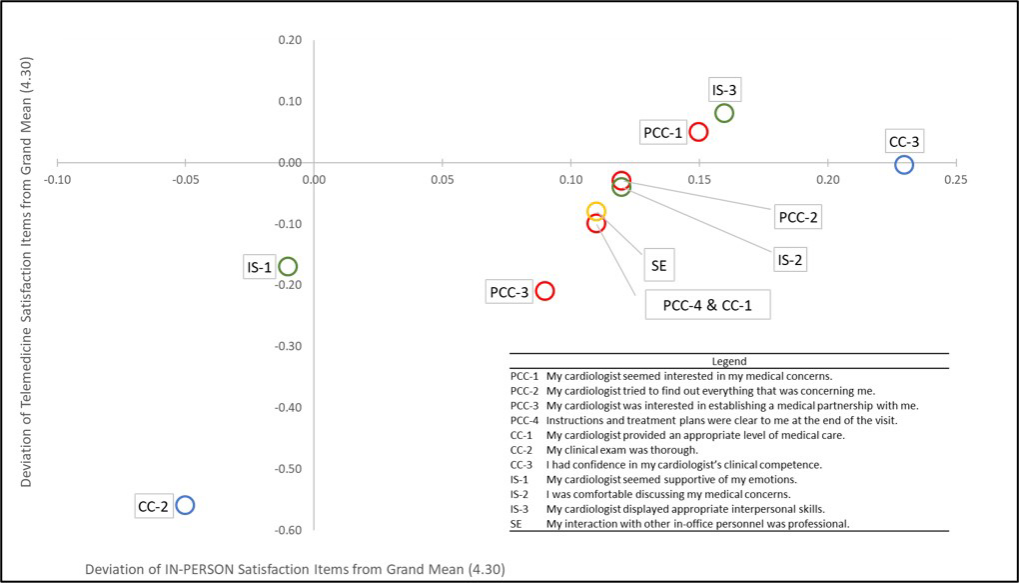
Changes in satisfaction ratings for individual survey items between telehealth and in-person. Points are expressed as the deviation of the mean of individual survey items from the grand mean of 4.30 for in-person (horizontal axis) and telehealth experiences (vertical axis). Labels reflect the survey domain and item number as indicated in Table 2. Points above and/or to the right of their respective axis indicate items whose mean rating was above the grand mean of all items, while those to the left and/or below indicate points with ratings below the grand mean. The lower right quadrant contains items for which in-person mean ratings were above the grand mean, while in telehealth were below the grand mean. CC: clinical competence; IS: interpersonal skills; PCC: patient-centered communication; SE: supportive environment.

Respondents rated the in-person experience somewhat higher across all 11 individual items (Table 2 and Figure 1); the mean rating in telehealth for 8 of the 11 items was below the grand mean, while only 2 items (patient-centered communication–1 and interpersonal skills–3) were above the grand mean; by contrast, only 2 items (clinical competence–2 and interpersonal skills–1) showed a mean in-person rating below the grand mean (Figure 1). The paired difference in average response was significantly lower for telehealth (*z*=3.98, *P*<.001). Despite this trend, only the item relating to the perceived thoroughness of the clinical exam showed a significantly different pattern of responses between appointment types. However, there was no significant difference in mean response to the single item related to respondents’ overall perception of their telehealth or in-person experience (*z*=1.22, *P*=.22). These data are summarized in Table 2.

All individual survey items showed significant positive correlations with respondents’ overall rating of their experience, across both telehealth and in-person formats, based on Bonferroni-adjusted criteria. For telehealth, Spearman correlations ranged from 0.49 for the item related to thoroughness of the clinical examination (*P*<.001) to 0.32 for the item related to the interaction with in-office personnel (*P*<.001). For the in-person experience, correlations ranged from 0.56 for the item relating to the clarity of instructions and treatment plans (*P*<.001) to 0.40 for the item related to the appropriateness of the level of medical care provided (*P*<.001). These data are summarized in Table 2.

**Figure 2:**
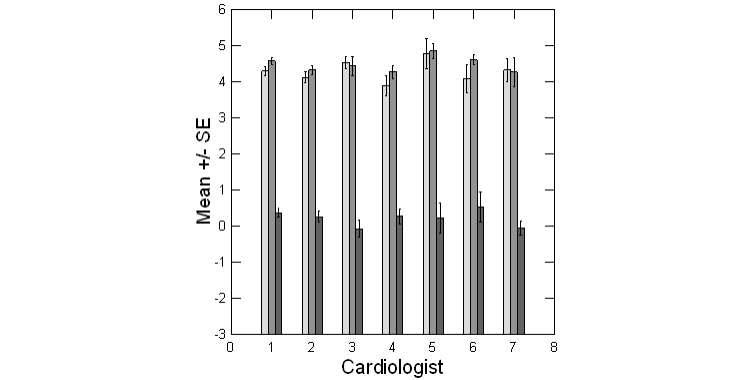
Average ratings of survey items relating to the telehealth (light grey bars) versus in-person experience (medium grey bars) by cardiologist. Dark grey bars represent the paired difference in ratings.

Average ratings for all cardiologists across both telehealth and in-person formats was uniformly high; all means for both were above 4.0 on a five-point scale (Figure 2). In addition, all cardiologists showed minimal difference in mean ratings across the two appointment types (Figure 2). There was no significant difference among cardiologists in their patients’ perceptions of either their telehealth (KW statistic 6.24, *df*=6, *P*=.40) or in-person experience (KW statistic 3.75, *df*=6, *P*=.71). Similarly, there was no difference among cardiologists in the paired difference in telehealth versus in-person ratings (KW statistic 7.2, *df*=6, *P*=.30).

### Perception: Advantages and Disadvantages to Telehealth

Reduced travel time was seen as a big advantage over traditional in-person appointments by 61 (57.5%) of the 106 respondents who participated in telehealth, and 94 (88.7%) viewed it at least somewhat of an advantage. Similarly, the majority (n=57, 53.8%) viewed reduced visit wait time as a big advantage, and 94 (88.7%) saw it as at least somewhat of an advantage. A similar percentage (n=87, 82.0%) saw travel cost savings as at least somewhat of an advantage to telehealth, including 43 (40.5%) who rated it as a big advantage. These data are summarized in Table 1. Respondents listed increased comfort, the ability to continue work, and lower risks of COVID-19 as additional benefits in the open response.

There was no relationship between communication modality (ie, phone, smartphone, computer, or tablet) and respondents’ overall rating of the telehealth experience (*χ*^2^_8_=6.14, *P*=.63); similarly, there was no relationship between software platform and overall ratings (*χ*^2^_2_=0.91, *P*=.63), though the majority of respondents (62.3%) indicated they did not remember the platform used. There was also no relationship between respondents’ travel time to in-person appointments and their overall rating of the telehealth experience (Spearman *r*=0.02, *df*=1, *P*=.24). Of 120 respondents, 100 (83.0%) indicated they would at least consider using telehealth in the future, including 59 (49.2%) who said they were likely to or would prefer to use telehealth going forward.

Among the 106 respondents who participated in telehealth, fewer than 10% (range 4-9 respondents, 3.8%-8.5%) rated any of the potential issues as a big disadvantage; by contrast, individual survey items were rated as *not a disadvantage* by 67%-86% (range 71-91) of respondents, based on their experience. Privacy concerns were seen as the least problematic of the potential issues, with only 15 (14.2%) respondents reporting this as at least somewhat of a disadvantage. Poor internet connectivity was of most concern, rated as at least somewhat of a factor by 35 (33.0%) respondents. These data are summarized in Table 1. Responses collected via open response included a lack of hands-on attention, difficulty communicating, and a lack of end-of-visit paper summaries as additional disadvantages.

## Discussion

### Access to Telehealth Offers Both Opportunities and Challenges

This study takes advantage of the natural experiment provided by the COVID-19 pandemic to explore the utility of telehealth from the patient perspective. We found both opportunities and challenges related to accessibility, and the modality is perceived by patients as a viable alternative to in-person office visits and patients saw clear benefits to its use. Our results have implications for cardiology practices moving forward but should be interpreted with caution due to sampling constraints and the unique context of the global pandemic.

Internet and technology access do not seem to be significant barriers to the use of telehealth. Of the 193 initial respondents, 55 (28.4%) reported declining to use telehealth. However, among the 32 respondents who declined and reported factors, only a small percentage (n=2, 6.2%) cited access to technology as a factor in their decision. Of the 106 respondents who participated in telehealth, a similarly low percentage (n=8, 7.5%) viewed internet connectivity as a big disadvantage, though a more substantial 25.5% (n=27) did cite it as somewhat of a disadvantage. Nevertheless, patients expressed a fairly high level of satisfaction with telehealth, in terms of both average ratings among items and overall rating of their experience. Similarly, more than 70% of respondents reported unfamiliarity with technology (both hardware and software) as not being a factor in declining telehealth or as a disadvantage by those who participated (n=82, 77.4% and n=76, 71.7%, respectively). These findings suggest that, even during a period of rapid and unplanned change, internet access and use of technology are likely manageable issues for most patients and that continued, intentional efforts on the part of governments, health care systems, and corporate providers to address access disparities will only improve the situation moving forward.

However, there is some evidence that it may be harder to coordinate telehealth appointments, at least initially. Although respondents did not indicate significant issues in navigating or communicating as part of their telehealth appointments, our data do suggest there was some ambiguity about the need for or opportunity to participate in telehealth. Of the 32 respondents who did not participate in telehealth, 27 (84.4%) cited not having an appointment as at least somewhat of a factor in their decision. However, all patients invited to participate in the study had an appointment scheduled with their cardiologist prior to the COVID-19–related executive orders prohibiting in-person delivery of nonacute health care services; these appointments were shifted to a telehealth format. The most common reason patients did not meet their telehealth appointment was inability of the WKHL office to contact patients the day of their appointment, and we suspect miscommunication between the WKHL office and the patients or patients’ family members regarding changes in the appointment modality as the possible reason for this. Going forward, it will be important for providers to ensure consistent and reliable communication with patients to minimize any confusion regarding appointments.

### Telehealth Is Perceived as a Viable Alternative to In-Person Cardiology Appointments

#### Patterns of Satisfaction Are Consistent Across Modalities

There was no significant shift in rankings of patient satisfaction scores between modalities. Although satisfaction scores decreased somewhat in telehealth for all items, the decreases were generally modest and consistent. This suggests that the different modalities do not present qualitatively different challenges to establishing a physician-patient relationship, though more intentional effort may need to be applied across the board to ensure that patients perceive telehealth as offering an equivalent standard of care.

#### Physicians Seem to Be Able to Adapt Well

Satisfaction scores were high and consistent among all 7 cardiologists represented in the sample. Despite having little or no previous experience with telehealth, all physicians appeared to operate effectively within the new environment. On a broader scale, there were few if any differences in patient satisfaction scores among the four survey domains of the physician-patient experience, both within and among telehealth and in-person modalities.

#### The Clinical Exam Is an Issue That Needs to Be Addressed

The only item that showed a significant decrease in patient satisfaction between in-person and telehealth visits was the perceived thoroughness of the clinical exam. Our patient population included a substantial number of older and rural individuals, many with limited technology abilities, limited access to technology, and limited access to broadband connection. This translated into a significant proportion of telehealth visits done without face-to-face evaluation, which might have contributed to a lower scoring on the physical examination component.

This finding is also consistent with existing concerns regarding telehealth in specialty fields [5]. It is clear that, if use of telehealth is to expand within cardiology or other similar fields, multiple mechanisms must be put in place to enable physicians to collect necessary clinical data remotely. Such remote patient monitoring solutions might include remote clinical stations located in partner clinics nearer to patients’ homes or use of smartphone apps that record heart rate, blood pressure, pulse oximetry, or electrocardiogram data and delivering those wirelessly to the physician [28].

### Patients See Clear Advantages to Using Telehealth

More than 80% of the 106 respondents identified time (n=94, 88.7%) and cost savings (n=87, 82.1%) as either somewhat or a primary advantage of telehealth, and overall satisfaction with telehealth was independent of the distance traveled by respondents to in-person appointments. This suggests that the perceived time and cost savings are threshold benefits that positively impact the majority of patients more or less equally. By contrast, privacy concerns were not viewed as a factor either by those who participated in telehealth or those who opted out. This pattern suggests that time and cost efficiency for patients should be a primary concern when implementing telehealth and that sensitive issues such as privacy protection can be readily accommodated.

### Limitations

Our study has some unavoidable limitations, due to its *natural experiment* dimension and the desire for real-time rapid response. The reliance on online delivery of the survey may well have limited our response rate, especially among those individuals less comfortable with or having limited access to technology. However, the ability to generate data on patient satisfaction in real time, as a means of rapidly assessing the mandated shift to telehealth, justifies its use. In any case, we appear to have captured a representative sample of our patient population, both demographically and in terms of accessibility (ie, telehealth no-shows), making the trends and relationships in our data worthy of further consideration. Moreover, post hoc power analyses indicated that our sample sizes were sufficient to establish a level of statistical power >0.95 for comparisons between telehealth and in-person visits.

Although our data highlight relevant lessons for the continued or expanded use of telehealth in cardiology, we must also be cautious. Satisfaction ratings of in-person appointments may be less reliable (and perhaps inflated) due to differences in reporting period; that is, we asked respondents to rate in-person experiences that occurred less recently than telehealth experiences. Longer reporting periods cause respondents’ ratings to be more affected by the most intense or recent experiences, while the impact of milder experiences is attenuated [29,30]. On the other hand, the dangers of COVID-19, especially for these patients who are at risk, nearly ensures a positive bias toward telehealth, which may disappear somewhat or entirely if and when the health care delivery system returns to more “normal” operation. As a result, we may have observed less difference between satisfaction with telehealth and in-person appointments than we might have originally expected (or might expect to see in the future). Once the fear related to the COVID-19 pandemic subsides, will patients still feel as positive about their experiences with telehealth?

These caveats suggest that, although we could expect the patterns among individual survey items to hold, we should be cautious in assuming that the degree of equivalency observed between telehealth and in-person satisfaction can be generalized to new health care delivery contexts. They also argue for considering even nonsignificant trends, as these may be indicative of differences that could become accentuated in a more normal environment. Finally, they highlight the need for randomized controlled trials to truly evaluate differences between in-person and telehealth experiences.

### Conclusions: Future Application of a Telehealth Model in Specialty Fields Such as Cardiology

The overall level of satisfaction expressed with telehealth and perceived time- and cost-saving benefits identified by patient indicate that it can play an increasing role in providing health care access and services beyond COVID-19, particularly in rural areas. As such, the efficacy of telehealth needs to be better examined, especially in medical specialty fields, and patient and provider perception of telehealth needs to be evaluated to determine if it is worth expanding into regular practice. Increased literature on telehealth use in rural populations will hopefully aid in determining the best course of action in addressing health care disparities in a substantial part of the United States.

## Abbreviations

EMR: electronic medical record
KW: Kruskal-Wallis
PACT: Patient Assessment of Communication During Telemedicine
WKHL: Western Kentucky Heart and Lung
